# Gene regulatory dynamics during the development of a paleopteran insect, the mayfly *Cloeon dipterum*

**DOI:** 10.1242/dev.203017

**Published:** 2024-10-10

**Authors:** Joan Pallarès-Albanell, Laia Ortega-Flores, Tòt Senar-Serra, Antoni Ruiz, Josep F. Abril, Maria Rossello, Isabel Almudi

**Affiliations:** ^1^ Department of Genetics, Microbiology and Statistics, Universitat de Barcelona, Diagonal 643, 08028 Barcelona, Spain; ^2^ Institut de Recerca de la Biodiversitat (IRBio), Universitat de Barcelona, Diagonal 643, 08028 Barcelona, Spain; ^3^ Institute of Biomedicine of Universitat de Barcelona (IBUB), Universitat de Barcelona, Diagonal 643, 08028 Barcelona, Spain

**Keywords:** Gene regulation, Paleoptera, Mayflies, Insects, Embryogenesis, ATAC-seq

## Abstract

The evolution of insects has been marked by the appearance of key body plan innovations that promoted the outstanding ability of this lineage to adapt to new habitats, boosting the most successful radiation in animals. To understand the evolution of these new structures, it is essential to investigate which genes and gene regulatory networks participate during the embryonic development of insects. Great efforts have been made to fully understand gene expression and gene regulation during the development of holometabolous insects, in particular *Drosophila melanogaster*. Conversely, functional genomics resources and databases in other insect lineages are scarce. To provide a new platform to study gene regulation in insects, we generated ATAC-seq for the first time during the development of the mayfly *Cloeon dipterum*, which belongs to Paleoptera, the sister group to all other winged insects. With these comprehensive datasets along six developmental stages, we characterized pronounced changes in accessible chromatin between early and late embryogenesis. The application of ATAC-seq in mayflies provides a fundamental resource to understand the evolution of gene regulation in insects.

## Teaser

**Summary:** New genomic resources using ATAC-seq reveal the dynamics of gene regulation during embryogenesis of the mayfly *Cloeon dipterum*, offering insights into insect development and evolution.

## INTRODUCTION

Insects make up the most numerous and diverse lineage of animals on the planet (Misof et al., 2014). This huge radiation has been possible due to their extraordinary capabilities to adapt to distinct environments, which resulted in more than thirty extant orders distributed worldwide (Grimaldi and Engel, 2005; Misof et al., 2014).

**Fig. 1.**
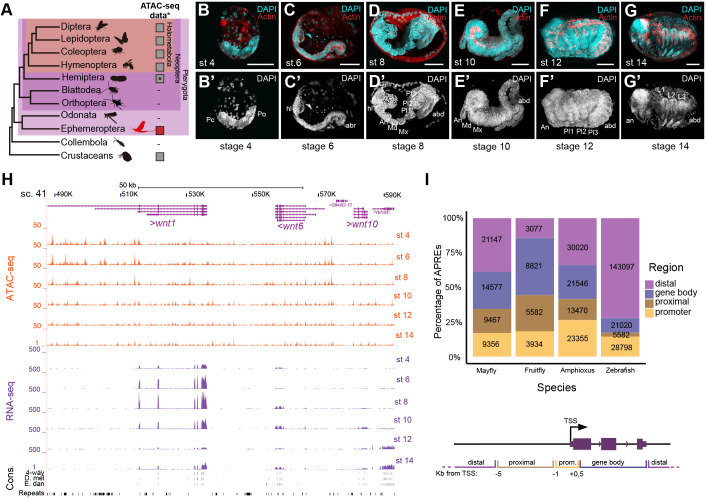
**Open chromatin profiles in *C. dipterum* embryogenesis.** (A) Simplified insect phylogeny. Grey squares highlight the availability of ATAC-seq datasets. Asterisk shows lineages in which there is no ATAC-seq information but there is FAIRE-seq material. (B-G′) Embryonic stages used in this study stained with Phalloidin-AlexaFlour-488 (Red) and DAPI (cyan). (B,B′) Stage (St) 4: germ band initial elongation. (C,C′) St6: S-shaped embryo. (D,D′) St8: Segmentation. (E,E′) St10: revolution or katatrepsis. (F,F′) St12: initial dorsalization. (G,G′) St14: dorsal closure completed. (H) UCSC genome browser showing *wnt1*, *wnt6*, *wnt10* cluster and the ATAC-seq tracks and RNA-seq tracks generated in this study and in Almudi et al. (2020). (I) Percentage of APREs distributed across *C. dipterum*, *D. melanogaster*, *B. lanceolatum* and *Danio rerio* (zebrafish) genomes. Scale bars: 50 µm.

This diversity of forms and lifestyles is the result of changes in the gene regulatory networks (GRNs) controlling the embryonic and postembryonic development of this clade (Carroll, 1998; Molina-Gil et al., 2023). In recent decades, the importance of regulatory information responsible for the location and time in which GRNs are functioning has been widely recognized (Andrikou and Arnone, 2015; Furlong and Levine, 2018; Gompel et al., 2005; Leyhr et al., 2022). These so called *cis* regulatory elements (CREs) are major players of morphological evolution not only in insects, but also in other animal lineages (Guerreiro et al., 2013; Leyhr et al., 2022). CREs are usually difficult to identify by homology, as they tend to accumulate more changes in their sequences that impede their proper characterization by sequence similarity (Zdobnov and Bork, 2007). The advent of new functional genomics approaches based on chromatin accessibility, such as formaldehyde-assisted isolation of regulatory elements (FAIRE-seq; Simon et al., 2012) and the Assay for Transposase-Accessible Chromatin (ATAC-seq; Buenrostro et al., 2013), has allowed the identification of open chromatin regions that can be assigned as CREs, such as enhancers, promoters and insulators. Therefore, much more comprehensive characterizations of CREs have been possible and several FAIRE-seq and ATAC-seq datasets have been recently generated to address distinct questions related to insect development and evolution. Nonetheless, these works have been mostly carried out using few established model species, such as the fruit fly *Drosophila melanogaster* (Diptera), the red flour beetle *Tribolium castaneum* (Coleoptera), some butterfly species (Lepidoptera) or certain species of ants and bees (Hymenoptera) (Lowe et al., 2022; Wang et al., 2020; Zhao et al., 2020), all of which belong to a single monophyletic group of insects, the Holometabola (Fig. 1A) (Schmidt-Ott and Lynch, 2016). By contrast, the remaining seventeen orders of winged insects (Fig. 1A) continue unexplored, with only adult and one embryonic FAIRE-seq datasets available (Fernández et al., 2020; Richard et al., 2017) to our knowledge.

All insect orders shared common phases during their development (Patel, 1994; Sander, 1976). Indeed, these main events are also present in non-insect arthropods (Zrzavý and Štys, 1997). The early stages of insect development initiate with several rounds of nuclear divisions that migrate to the periphery to form the blastoderm. The blastoderm gives rise to different types of germ band (short, intermediate or long) that are subsequently segmented during the following embryonic stages (Davis and Patel, 2002). Then, the segmented embryo undergoes a process of differentiation in which organogenesis and the final development of the juvenile structures take place (Sander, 1976).

The mayfly *Cloeon dipterum*, a recently established laboratory system (Almudi et al., 2019, 2020), is in a privileged position to improve the phylogenetic diversity of insect functional genomics resources. Mayflies or Ephemeroptera belong to the Paleoptera group of winged insects, together with Odonata (dragonflies and damselflies) (Fig. 1A) (Simon et al., 2018). They are the sister group to all other winged insects and, thus, they are key to address fundamental questions related to insect ecology, development and evolution.

Here, we performed ATAC-seq experiments at six different developmental stages in *C. dipterum* embryos. Our identified accessible chromatin regions provide an exhaustive collection of putative promoters and enhancers along embryogenesis of this insect. Moreover, by studying the temporal dynamics of these elements, we showed wholesale changes in chromatin accessibility during the transition between the last stages of segmentation and the start of organogenesis. Finally, we facilitate the access to these comprehensive datasets through a dedicated UCSC Genome Browser Hub (https://genome-euro.ucsc.edu/s/mayfly/Clodip), providing a key resource available for the entire community to understand the evolution of gene regulation during the development of winged insects.

## RESULTS AND DISCUSSION

### Open chromatin profiles in *C. dipterum* embryogenesis

To investigate dynamics of gene regulation during the embryogenesis of mayflies, we performed ATAC-seq assays for six different developmental stages: stage (St) 4 (germ band elongation, Fig. 1B,B′), St6 (S-shaped embryo: anatrepsis II, Fig. 1C,C′), St8 (segmentation of the embryo, Fig. 1D,D′), St10 (revolution: katatrepsis, Fig. 1E,E′), St12 (start of dorsal closure, Fig. 1F,F′), St14 (dorsal closure complete, Fig. 1G,G′) (Tojo and Machida, 1997). Sequences resulting from these experiments were mapped against the *C. dipterum* reference genome assembly (CLODIP2; Almudi et al., 2020) (see Materials and Methods and Fig. S1) to obtain a non-redundant collection of open chromatin regions throughout the genome that we termed APREs (Accessible Putative Regulatory Elements) (Fig. 1H). After normalization (see Materials and Methods) we identified a total of 54,547 APREs across the six stages. Of them, 45,649 APREs did not show changes in accessibility in our clustering analyses of the different developmental samples we assayed (i.e. they remained constitutively open or closed across these stages), whereas 8898 APREs were dynamic and changed their accessibility across stages (Fig. S2 and Table S1).

We next aimed at defining the genomic distributions of the APREs relative to genes and gene annotations. For this, we calculated the proportions of APREs at the ‘promoter’ [i.e. APREs in the immediate vicinity of the annotated transcription start sites (TSSs)], at ‘proximal regions’, spanning up to 5 kb upstream the TSSs, at ‘gene bodies’ (located between the end of the promoter and the termination site of the gene) and at ‘distal’ regions, which comprised regions that do not fall in the previous categories (Fig. 1I, Table S2). We found that both non-dynamic and dynamic APREs were distributed in similar proportions (Fig. S3) and only detected a slight increase in non-dynamic APREs located in promoters with respect to dynamic APREs in promoters (∼18% versus ∼13%) and an even slighter difference between non-dynamic and dynamic APREs in gene bodies (26% and 29%, respectively, Fig. S3). This proportion of APRE distribution was similar to the distribution of APREs in other invertebrate genomes, such as the chordate amphioxus (*Branchiostoma lanceolatum*) (Marlétaz et al., 2018). These two invertebrate species, *C. dipterum* and *B. lanceolatum*, notably diverged from the distribution found in some vertebrate species, which showed a much larger proportion of distal APREs due to the relevance of distal regulation in these vertebrate genomes and the impact of the different rounds of whole genome duplications (Marlétaz et al., 2018). By contrast, when we compared the APRE distribution of *C. dipterum* with APRE distribution in *D. melanogaster*, we observed that fruit flies had a much lower proportion of distal APREs (i.e. a third of the corresponding fractions in *C. dipterum* and *B. lanceolatum*), with a higher proportion of APREs located in gene bodies and proximal regions (Fig. 1I) (Bozek et al., 2019). These differences between *C. dipterum* and *D. melanogaster* were most likely due to the higher compaction of the 120 Mb *D. melanogaster* euchromatic genome (Adams et al., 2000), where most of the ancestral genomic regulatory blocks and their associated long range regulatory interactions have been dismantled (Irimia et al., 2012).

### ATAC-seq revealed a temporally regulated chromatin profile in the mayfly genome

To study changes in chromatin accessibility throughout different samples (i.e. developmental time points), we analyzed differential APRE activity between consecutive developmental stages. These results showed relatively modest changes between successive time points (i.e. 164 APREs at the St4-St6 transition or 365 at St10-St12; Fig. S4, Table S3), with the exception of the transition between St8 and St10, when 4568 APREs changed their accessibility, from 7.5 to 27 times more than at the other time points (Fig. S4). From these, the vast majority (3118) corresponded to APREs that increase accessibility during this St8-St10 phase. This large amount of differentially active APREs between these two stages may indicate a major regulatory turnover between an early and late regulatory state during mayfly embryogenesis. In fact, this major shift was also evident when we performed a principal component analysis (PCA) analysis and clustering of the ATAC-seq datasets, which formed two very distinctive clusters: samples from stages 4, 6 and 8 and samples from 10, 12 and 14 (Fig. S4).

**Fig. 2.**
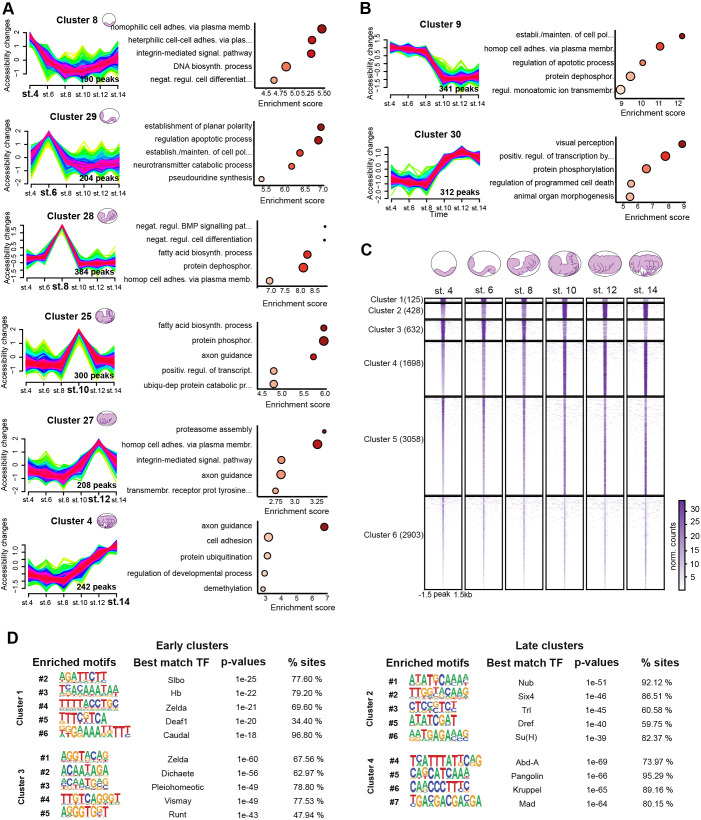
**ATAC-seq revealed temporally regulated chromatin profile in the mayfly genome.** (A) Mfuzz clusters obtained for the 8898 dynamic APREs representing stage-specific APRE activity and associated GO enriched terms (dot size: number of significant observations; color fill: values as on *y*-axis). (B) Mfuzz clusters representing ‘early’ and ‘late’ embryogenesis APRE activity and their associated GO enriched terms. (C) Heatmaps of the 8898 dynamic APREs clustered using k-means clustering. Six clusters were obtained, with four of them showing a clear dynamic behavior: cluster 1 (*n*=125) and cluster 3 (*n*=632) early activity, cluster 2 (*n*=428) and cluster 4 (*n*=1698) late activity. (D) Motif enrichment of early (clusters 1, 3) and late (clusters 2, 4) active clusters. Five or four representative motifs of the top-10 are shown. Motif logos are represented with their position in the top 10, TF names, enrichment *P*-values and percentage of sites were obtained from the HOMER suite (see Material and Methods).

To further explore the dynamics of chromatin accessibility across the selected timepoints, we performed a temporal soft-clustering analysis using Mfuzz (Kumar and Futschik, 2007) (see Materials and Methods and Table S4). Among the different temporal clusters obtained (Fig. S5), we focused on clusters of APREs for which accessibility peaked at single embryonic stages (e.g. clusters 8 and 16 for St4, clusters 29 and 21 for St6, clusters 28, 5, 6, 11, 13, 14 and 26 for St8, clusters 25, 15 and 18 for St10, cluster 27 for St12 and clusters 4 and 2 for St14; Fig. 2A and Fig. S5). We then associated these APREs to their putative target genes and analyzed the Gene Ontology (GO) enriched terms for each of these stage-specific clusters, using *D. melanogaster* orthologs (Almudi et al., 2020), as it is the closest organism with functional annotation available (see Materials and Methods and Table S5). This GO term enrichment analysis exhibited categories highly connected with each of the embryonic stages in which the accessibility of the chromatin was higher (Fig. 2A and Table S5). For example, cluster 8 (St4) and cluster 29 (St6) contained APREs that were associated with genes involved in cell adhesion, DNA biosynthetic process and negative regulation of cell differentiation, which are characteristic processes of early embryogenesis in insects (Brantley and Di Talia, 2021; Münster et al., 2019). On the other hand, GO terms enriched in clusters corresponding to later stages of embryogenesis (e.g. clusters 25, 27 and 4; Fig. 2A and Table S5) revealed processes related to organogenesis, such as axon guidance or regulation of developmental process (Gillott, 2005).

Besides these stage-specific clusters, we also found several clusters that showed more prolonged activity patterns. In this manner, we identified clusters that recapitulated the major developmental shift we had previously observed between St8 and St10, with a set of ‘early embryogenesis’ clusters (e.g. clusters 7, 9, 17, 19, 21 or 26) and another of ‘late embryogenesis’ ones (clusters 3, 22, 23 or 30; Fig. 2B and Fig. S5). Accordingly, we identified enriched GO terms related to early development (e.g. establishment and maintenance of cell polarity or cell adhesion processes) and terms related to late embryogenesis (e.g. visual perception and animal organ morphogenesis), respectively (Fig. 2B and Table S5).

In addition, we also performed *k-means* hard clustering (see Materials and Methods; Table S6) using the same set of dynamic APREs (Fig. 2C). This analysis was able to recover six clusters with differential dynamics of accessibility, although none of these clusters corresponded to APREs showing stage-specific accessibility. By contrast, we identified two groups of clusters – clusters 1 and 3 on one hand, and clusters 2 and 4 on the other hand – that contained APREs accessible during early stages of embryogenesis and late stages of embryogenesis, respectively (Fig. 2C and Table S6), mirroring the St8-St10 shift observed in the previous analyses. To better characterize these clusters, we carried out transcription factor (TF) motif enrichment analysis using Homer software (see Materials and Methods; Fig. 2D and Table S7). In agreement with previous work in other insects and the results of the GO enrichment analysis from Mfuzz clusters (Fig. 2A,B), APREs in clusters 1 and 3 showed motifs with a best match of TFs involved in early embryogenesis. These include Hunchback (Hb), involved in antero-posterior axis specification (Qian et al., 1991), Caudal (Cad), which functions in germ band elongation (Schulz and Tautz, 1995; Wu and Lengyel, 1998), or Zelda (Zld), a zygotic genome activator that acts during early blastoderm development (Brennan et al., 2023; Liang et al., 2008) (Fig. 2D). By contrast, clusters 2 and 4, with APREs that were open in later stages of development, showed enrichment in motifs that correspond to TFs involved in different processes of organogenesis, such as Nubbin (Nub), a regulator of appendage morphogenesis (Turchyn et al., 2011), or Mothers against dpp (Mad), which mediates the response to the BMP pathway during the development of diverse insect organs (Sekelsky et al., 1995).

Overall, these analyses revealed two main phases during the embryogenesis of mayflies in which distinct set of regulatory regions are active (Fig. 2B,C) to control different sets of GRNs involved in such early or late embryonic processes. These results were consistent with the mid-developmental transition previously described at transcriptomic level for some phyla, including insects (Levin et al., 2016).

### Chromatin accessibility to understand gene expression dynamics

**Fig. 3.**
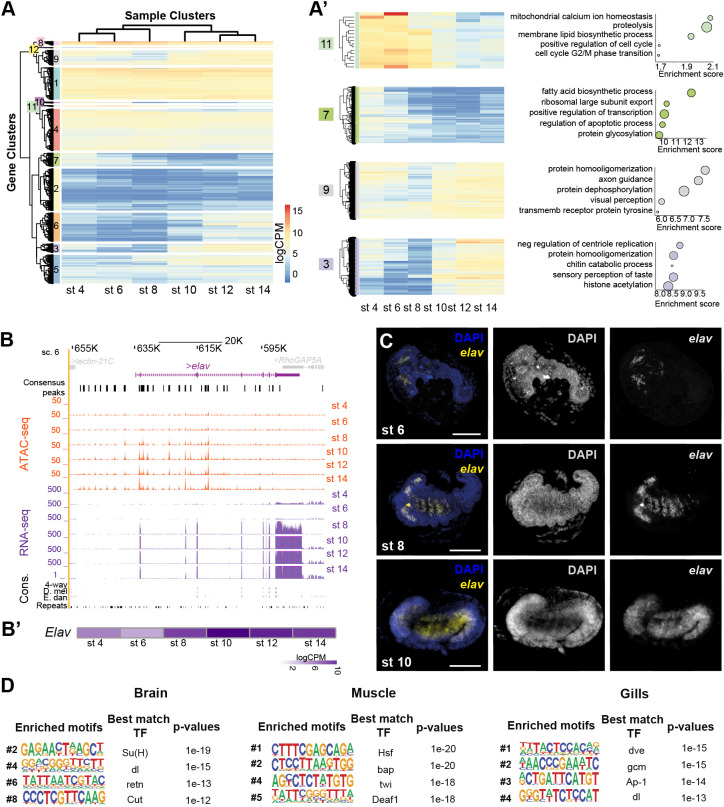
**Chromatin accessibility to understand gene expression dynamics.** (A) Heatmap showing expression levels of genes associated with dynamic ATAC-seq APREs. RNA-seq samples clustered according to embryonic stage progression. Secondary clustering showed 12 different gene clusters. (A′) Selected gene clusters with the associated GO terms (dot size: number of significant observations). (B) *elav* genomic regulatory landscape. APRE activity and gene expression increase as embryogenesis progresses. (B′) Heatmap of the *elav* expression across embryonic stages. (C) HCR hybridization against *elav* at stages 6, 8 and 10 of embryogenesis. Nuclei were stained with DAPI (blue) and *elav* expression is shown in yellow. Scale bars: 50 µm. (D) Motif enrichment of APREs associated with genes from tissue-specific WGCNA modules identified in Almudi et al. (2020). Four representative motifs of the top 10 were chosen. Motif logos are represented with their position in the top 10, TF names and enrichment *P*-values were obtained from the HOMER suite (see Material and Methods).

As ATAC-seq has been proven to be a powerful method to investigate the regulation of gene expression, we next addressed the relationship between chromatin accessibility and levels of gene expression (Starks et al., 2019). To do this, we measured the levels of gene expression at the same developmental stages of our ATAC-seq datasets. We focused on genes associated to the 8898 dynamic APREs and clustered them using Euclidean distances (see Materials and Methods; Fig. S6 and Table S8). We first observed that these genes clustered according to the six developmental stages and the ‘early’ and ‘late’ embryonic phases that we identified in the ATAC-seq data. Transcriptomes from stages 4, 6 and 8 formed a cluster, whereas transcriptomes from stages 10, 12 and 14 grouped together (Fig. 3A). Moreover, we detected some genes with an expression that varied along the developmental time points we characterized: clusters 11 and 7 decreased their expression as embryogenesis progressed, whereas genes from clusters 3, 5 or 9 increased their expression during embryonic development (Fig. 3A,A′). GO terms enrichment analysis of these 12 gene clusters showed that the genes primarily expressed during early embryogenesis (clusters 11 and 7) were terms related to cell cycle progression and biosynthesis (e.g. positive regulation of cell cycle). Conversely, the genes that showed increased expression in late embryogenesis (clusters 9 and 3) are associated with organogenesis (e.g. axon guidance) (Fig. 3A′, Fig. S6C and Table S9). To further illustrate our results, we investigated the expression pattern of *embryonic lethal abnormal vision* (*elav*) that showed differential APRE accessibility and differential gene expression along embryonic stages (Fig. 3B,B′). *Elav* is an RNA binding protein involved in axon guidance, synapse formation and development of neurons (Robinow and White, 1988). We performed Hybridization Chain Reaction (HCR) assays (https://hackmd.io/@ColbyMBL/hcr) in St6, St8 and St10 embryos to characterize the spatial expression of this gene. Although embryos at St6 showed reduced expression of *elav* in some cells in the cephalic region (Fig. 3C), at St8 *elav* exhibited a broader expression domain in head domains and in the most anterior thoracic segments. At St10, these neural territories of *elav* expression elongated to the abdominal segments (Fig. 3C). As expected, we also observed an increase in the accessibility of chromatin in the locus, especially upstream of the TSS (Fig. 3B).

Previous analysis of co-regulated gene expression across several tissues, using Weighted Gene Correlation Network Analysis (WGCNA), revealed modules of genes specifically expressed in particular adult and nymphal tissues (Almudi et al., 2020). We examined whether chromatin accessibility information from embryos correlated with these modules. We characterized enriched motifs in APREs associated to the genes contained in each of these WGCNA modules and found distinctive enriched binding motifs for some of them (see Materials and Methods and Table S10). For example, the brain module was enriched in neural motifs, such as Suppressor of Hairy [Su(H)], retained (retn) or Cut (Ct), which are TFs involved in neural or glial development (Grueber and Jan, 2004; Shandala et al., 1999), the muscle module had motifs for bagpipe (bap) or twist (twi), required for mesodermal development (Castanon et al., 2001; Cripps and Olson, 2002) and specification, or the gills module with enrichment in motifs such as defective proventriculus (dve) involved in epithelium patterning or glial cells missing (gmc), which could have a role in the determination of some of the neural cells described in these abdominal structures (Almudi et al., 2020; Hosoya et al., 1995) (Fig. 3D and Table S10). These results suggest that some of the GRNs involved in the development of nymphal tissues and organs are already functioning during embryogenesis and their regulatory signatures can be detected in our ATAC-seq datasets. Thus, our results can also provide important insights into the regulatory logic of the adult body plan, and therefore also constitute a valuable resource for adult insect biology.

Overall, our ATAC-seq datasets provide a comprehensive resource to help uncover developmental diversity of insects, as it represents the first publicly available genome-wide collection of putative regulatory elements across embryogenesis in a paleopteran lineage using ATAC-seq. Thus, the key phylogenetic position of Ephemeroptera, together with the extensive chromatin accessibility information made available here, will open new avenues to address longstanding questions in the fields of developmental and evolutionary biology and comparative genomics.

## MATERIALS AND METHODS

### Culture maintenance, embryo collection and fixation

Samples were obtained from a *C. dipterum* culture maintained in the laboratory as previously described in Almudi et al. (2019). Gravid females fertilized on different days were collected and dissected to obtain embryos at selected developmental stages: St4, St6, St8, St10, St12 and St14. After opening the abdomen of these gravid females, embryos were collected to perform ATAC-seq or RNA-seq procedures and a small subset was collected separately and fixed with 4% formaldehyde for 1 h at room temperature (RT) to confirm the developmental stage. After 3× 5 min washes with PBS, these fixed embryos were stained with Phalloidin Alexa Fluor™ 488 (A12379, Thermo Fisher Scientific) and DAPI to visualize actin filaments and nuclei, respectively. Images were acquired using a Zeiss LSM 880 confocal microscope and were processed with Fiji (Schindelin et al., 2012).

### HCR hybridization

HCR hybridization followed a modified version of the Molecular Instruments HCR v.3 protocol (https://hackmd.io/@ColbyMBL/hcr). The HCR probe was designed to evade non-specific binding using an open-source probe design program (Kuehn et al., 2022). Briefly, embryos stored in ethanol were rehydrated in an ethanol series (75%, 50%, 25%) in PBTw [phosphate-buffered saline (PBS), 0.1% Tween 20] 0.1%. After 3× 5 min washes in PBTw 0.1%, embryos were permeabilized in detergent solution [1.0% SDS, 0.5% Tween, 50.0 mM Tris-HCl (pH 7.5), 1.0 mM EDTA (pH 8.0) and 150.0 mM NaCl] for 30 min at RT, kept in pre-warmed Probe Hybridization Buffer (Molecular Instruments) for 30 min at 37°C, and incubated in Probe Solution (4 nM of probe in Probe Hybridization Buffer) overnight at 37°C. After 4×15 min washes in pre-heated wash buffer (Molecular Instruments) at 37°C and 2×5 min washes in 5× SSCTw [saline sodium citrate (SSC), 0.1% Tween 20] 0.1% at RT, they were kept in pre-equilibrated Amplification Buffer (Molecular Instruments) for 30 min at RT and incubated in hairpin solution [60 nM of each hairpin h1 and h2 (Molecular Instruments) separately in pre-equilibrated Amplification Buffer], heated at 95°C for 90 s and cooled down for 30 min overnight in the dark at RT. Following 5×20 min washes in 5× SSCTw 0.1% and 1×10 min wash in PBTw 0.1% pH 7.4 in the dark at RT, embryos were mounted in Prolong™ Gold with DAPI (P36941, Invitrogen). Images were acquired using a Zeiss LSM 880 confocal microscope and were processed with Fiji (Schindelin et al., 2012).

### RNA-seq and assembly

Three RNA-seq datasets (including replicates) of St8 and St12 embryos were generated using the Illumina technology. Samples were processed immediately after dissection and RNA was extracted using RNeasy Mini Kit (Qiagen) following the manufacturer's instructions. Paired-end libraries were generated using Illumina (Novaseq6000) 2×50 bp. After quality control, the obtained reads were aligned using the STAR aligner. Initially, a genome index was created using the CLODIP2 reference genome (GCA_902829235.1) with the genomeGenerate mode of STAR. Subsequent alignment of reads to this index was performed using the alignReads mode. Gene expression levels were quantified using the quantMode GeneCounts option within STAR.

### ATAC-seq and library preparation

ATAC-seq from Buenrostro et al. (2015) was optimized during these experiments to use on mayflies. Briefly, embryos were homogenized in lysis buffer (10 mM Tris-HCl pH 7.4, 10 mM NaCl, 3 mM MgCl_2_, 0.1% NP-40) to obtain ∼70,000 individual nuclei. After removing lysis buffer, a transposition reaction (1.25 μl of Tn5 enzyme in 10 mM Tris-HCl pH 8.0, 5 mM MgCl_2_, 10% w/v dimethylformamide) was performed for 30 min at 37°C and the resulting fragments were purified using MinElute PCR Purification Kit (Qiagen). Quantitative PCR was performed to determine the optimal number of cycles necessary for each library. A unique pair of primers were assigned to each sample (Table S11) and 12 libraries were prepared corresponding to two biological replicates of the six selected developmental time points using a PCR. Libraries were purified using the MinElute PCR Purification Kit (Qiagen). DNA concentration in each sample was calculated with Invitrogen™ Qubit™ 4 Fluorometer using the Qubit 1× dsDNA HS Assay Kit.

### ATAC-seq mapping and peak (APRE) calling

For peak (APRE) calling, we followed the pipeline outlined in https://github.com/alexgilgal/Thesis_methods/tree/main/ATAC-seq%20analysis, implementing minor modifications as detailed in our project's GitHub repository (https://github.com/mayflylab/Cdip-RegEmb/tree/main). We employed the ATAC_pipe.pl script for mapping reads, using Bowtie2 (Langmead and Salzberg, 2012) to align the reads to the CLODIP2 reference genome (GCA_902829235.1). Following alignment, the resulting BAM files were filtered based on a quality threshold of 10 and a minimum fragment length of 130 bp (Fig. S1).

The processed files were then subjected to peak analysis using the idr_ATAC_script.sh script, which executes peak calling with MACS2 (Zhang et al., 2008) to generate two sets of peaks: conservative peaks, indicating high-confidence regions across biological replicates, and optimal peaks, denoting reproducible events that consider read sampling variability, derived from pseudo-replicates. Subsequent IDR (Li et al., 2011) analysis was performed on both peak sets. Peak statistics – including the number of peaks and rescue ratios – were calculated and documented in a summary file.

### APRE classification and gene assignment

APREs were classified and associated with genes based on their proximity to the TSSs. TSSs were defined using the get_TSS.py script (https://github.com/m-rossello/GeneRegLocator/). To classify APREs and link them to genes, we used a custom-made script named make_table_from_zones.py (https://github.com/m-rossello/GeneRegLocator/). This script is designed to delineate regulatory zones around TSSs and associate these zones with APREs from ATAC-seq data. It defines three types of regulatory zones: promoters, located near the TSS, spanning 1000 bases upstream and 500 bases downstream; proximal regions, positioned further from the TSS, extending 4000 bases upstream but not overlapping with promoters; gene bodies, encompassing regions within the gene but excluding the promoter areas (Fig. 1I). Zones are non-overlapping on the same strand, although the same genomic position can exhibit different zones on each strand. Each APRE was associated with one or more genes if it overlapped by more than 70% with a gene zone. APREs not falling within promoter, proximal or gene body regions were classified as distal and remained unassociated with any gene (Table S2).

### Open chromatin analysis

The counts obtained from consensus APREs were used for all subsequent analyses, following normalization. This normalization involved adjusting the count data using the TMM method (Robinson and Oshlack, 2010) to account for differences in library size and composition. To further explore trends and variations across different biological stages, we aggregated the normalized counts by these stages, calculating mean values. Global APRE analysis categorized APRE as either ‘open’ or ‘closed’ based on a threshold of ten counts. APREs registering fewer than ten counts were deemed closed. We defined non-dynamic APREs as those that remained consistently open or closed across all examined stages. Conversely, dynamic APREs were characterized by their variability, changing between open and closed states across different stages or samples.

### Differential chromatin accessibility analysis

After counts normalization by TMM (Robinson and Oshlack, 2010) and sample exploration by PCA and clustering, differential chromatin accessibility analysis was performed in dynamic APREs. For this analysis, we used the limma-trend method (Law et al., 2014; Phipson et al., 2016). This approach was applied to the normalized count data of dynamic APREs to identify significant differences in chromatin accessibility between conditions. To visualize the results, we generated volcano plots using the EnhancedVolcano package (https://github.com/kevinblighe/EnhancedVolcano).

### Mfuzz analysis

We conducted the Mfuzz cluster analysis using the mfuzz function from the R package Mfuzz (Kumar and Futschik, 2007). Dynamic APREs with mean values computed by developmental stage were analyzed. The optimal parameters were systematically determined, setting the fuzzifier value at m=1.5 and the number of clusters at 30.

### k-means analysis

We performed k-means hard clustering using the DeepTools package (Ramírez et al., 2016) to analyze dynamic APRE enrichments from ATAC-seq data. This analysis included computing genome region scores with the computeMatrix function. The generated matrices enabled the visualization of heatmaps, which provided insights into the distribution of dynamic APREs across various developmental stages.

### Gene ontology enrichment analysis

For our GO enrichment analysis, GO annotations were transferred from the UniProt proteome (UP000494165) to the genes associated with the APREs. We performed statistical analysis using the topGO package (https://bioconductor.org/packages/release/bioc/html/topGO.html), which uses the elimination algorithm to identify significantly enriched GO terms within gene clusters. The BH false discovery rate correction method (Benjamini and Hochberg, 1995) was employed to control multiple testing. The results were visualized using GO enrichment plots, created with the ggplot2 package.

### TFBM enrichment analysis

Transcription factor binding motif (TFBM) enrichment analysis was conducted using the findMotifsGenome.pl tool from the HOMER suite (Heinz et al., 2010). We designated the APREs of interest as the foreground and used the remainder of the consensus APREs as the background. The fragment size selected for motif discovery corresponded precisely to the regions of the APREs (-size given). This analysis included a comparison against collected motifs specific to insects.

### RNA-seq analysis

For this analysis, we used the RNA-seq data generated in this project for stages 8 and 12. The remaining stages were obtained from publicly available data previously generated in the lab (PRJEB35103). Raw counts were obtained using STAR as previously described. To reduce batch effects, we normalized the data using the TMM method from EdgeR (Robinson and Oshlack, 2010) and transformed the counts to logCPM.

Heatmaps were created using the pheatmap function from the pheatmap package (Pretty Heatmaps, R package version 1.0.12; https://github.com/raivokolde/pheatmap). Clustering was performed using Euclidean distance.

### Use of AI tools

In the preparation of this manuscript, the AI tool ChatGPT-4 was used for assistance in specific areas. ChatGPT-4 was employed to detect errors in the code and to provide code suggestions. However, all underlying ideas and logic of the code are entirely original and were developed independently by the authors.

The use of AI tools has been conducted responsibly, and the authors take full accountability for the content of this manuscript, including the sections influenced by AI assistance.

## Supplementary Material



10.1242/develop.203017_sup1 Supplementary information

Table S1. Counts to all the identified consensus APREs before and after normalization and mean aggregation per stage.

Table S2. APREs associated to each gene and genomic zone.

Table S3. Differential APRE activity data

Table S4. Mfuzz output values.

Table S5. Gene ontology enrichment for each Mfuzz cluster.

Table S6. APREs in each cluster in the kmeans clustering.

Table S7. Homer motive enrichment results for each kmeans cluster.

Table S8. RNA counts before and after normalization.

Table S9. Gene ontology enrichment for each gene cluster.

Table S10. Homer motive enrichment results for each WGCNA module.

Table S11. List of primers used for library preparation.
