# Supplementary material for: Gene regulatory dynamics during the development of a paleopteran insect, the mayfly Cloeon dipterum
Source: Development. 2024 Oct 10;151(20):dev203017. doi: 10.1242/dev.203017 (PMC11491810; doi:10.1242/dev.203017)
Supplement: Supplementary information [file develop-151-203017-s1.pdf]

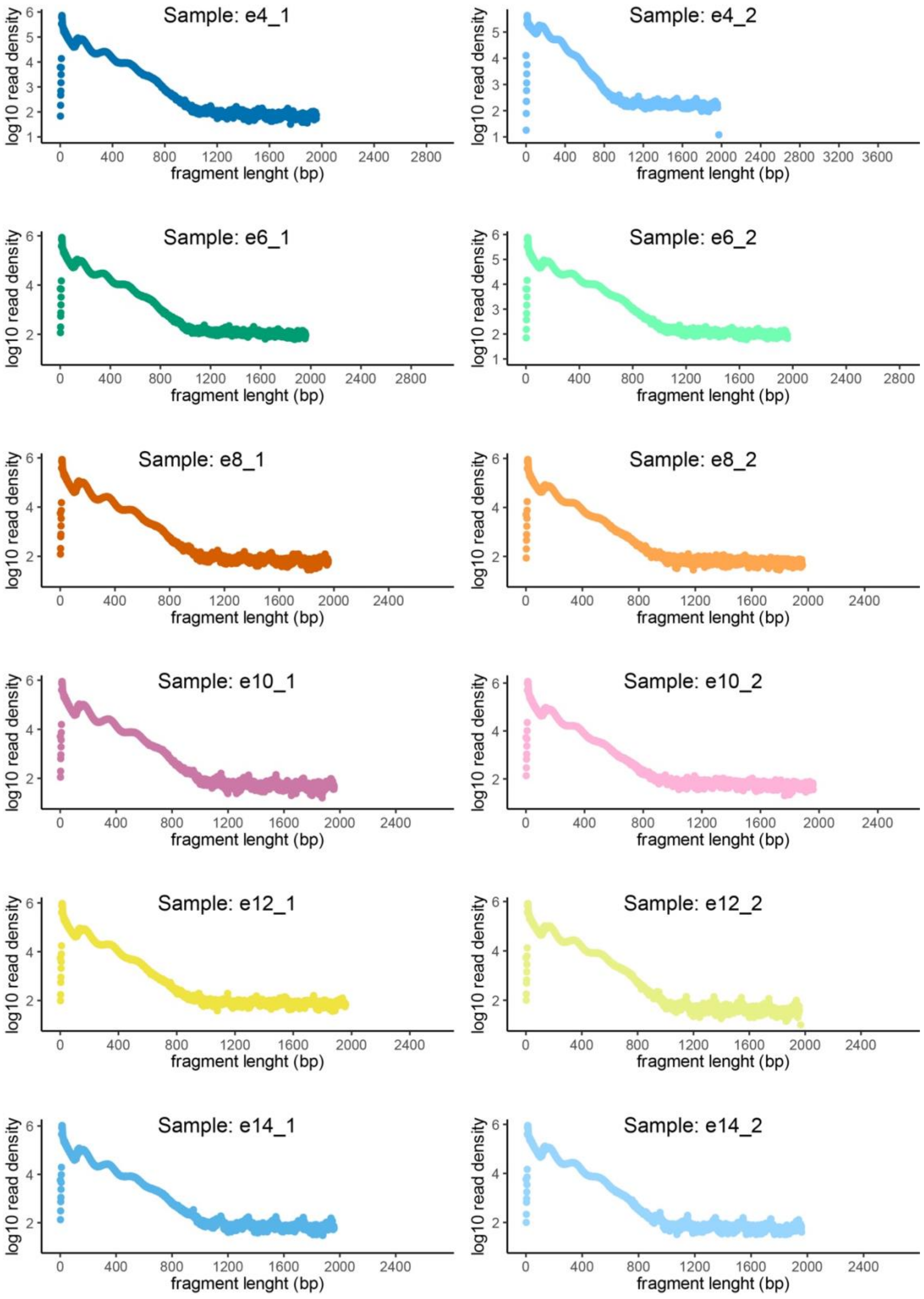

Fig. S1. Read size distribution in ATAC-seq libraries.

Development • Supplementary information

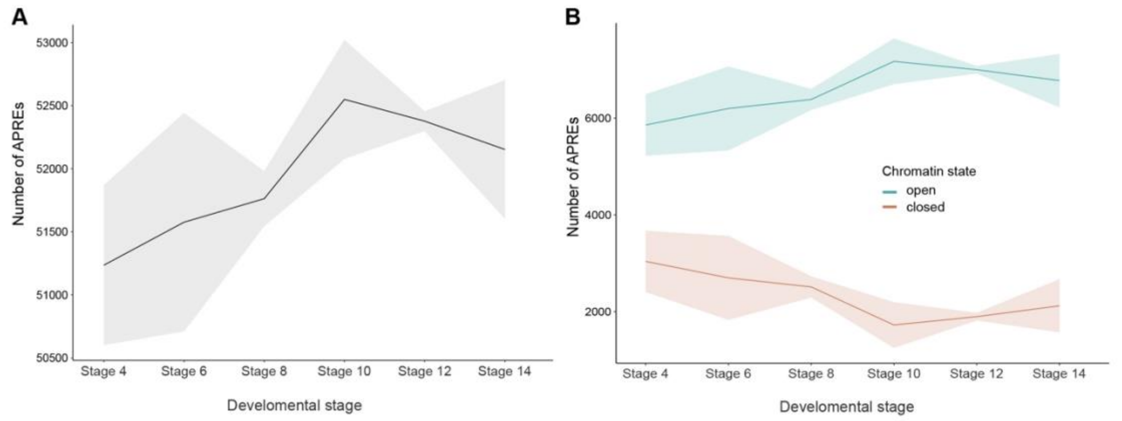

Fig. S2. Chromatin state across different developmental stages. (A) Total number of APREs identified as open at each developmental stage. **(B)** Chromatin APREs dynamically opening and closing across the stages.

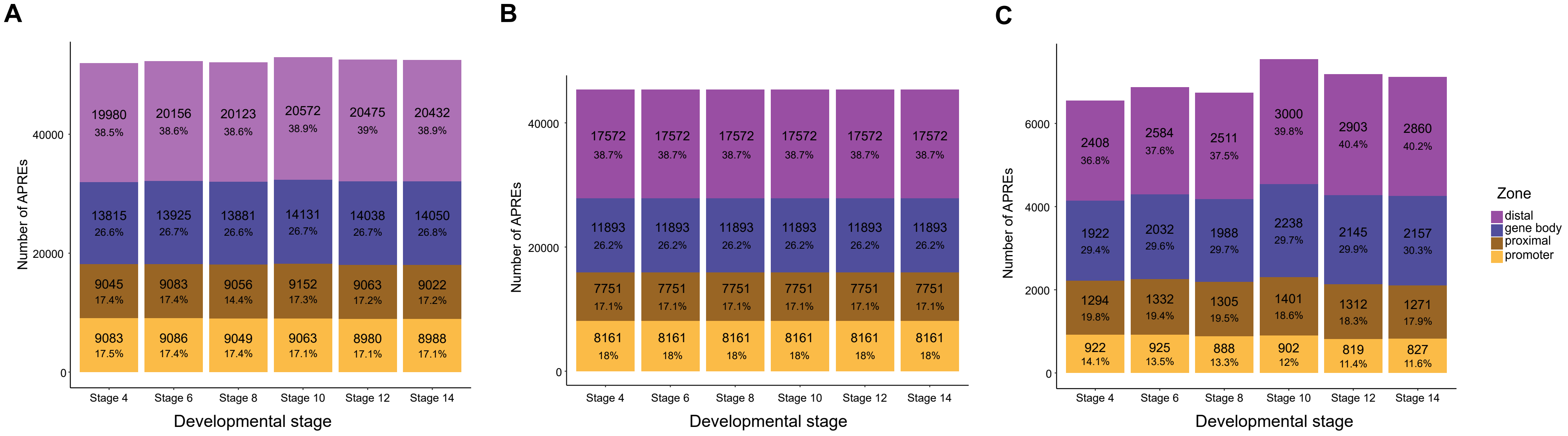

Fig. S3. Distribution of APREs per developmental timepoint. (A) Number of APREs in each genomic zone distributed across each developmental stage. (B) Non-dynamic APREs per genomic zone distributed across each stage. (C) Dynamic APREs per genomic zone distributed across each stage.

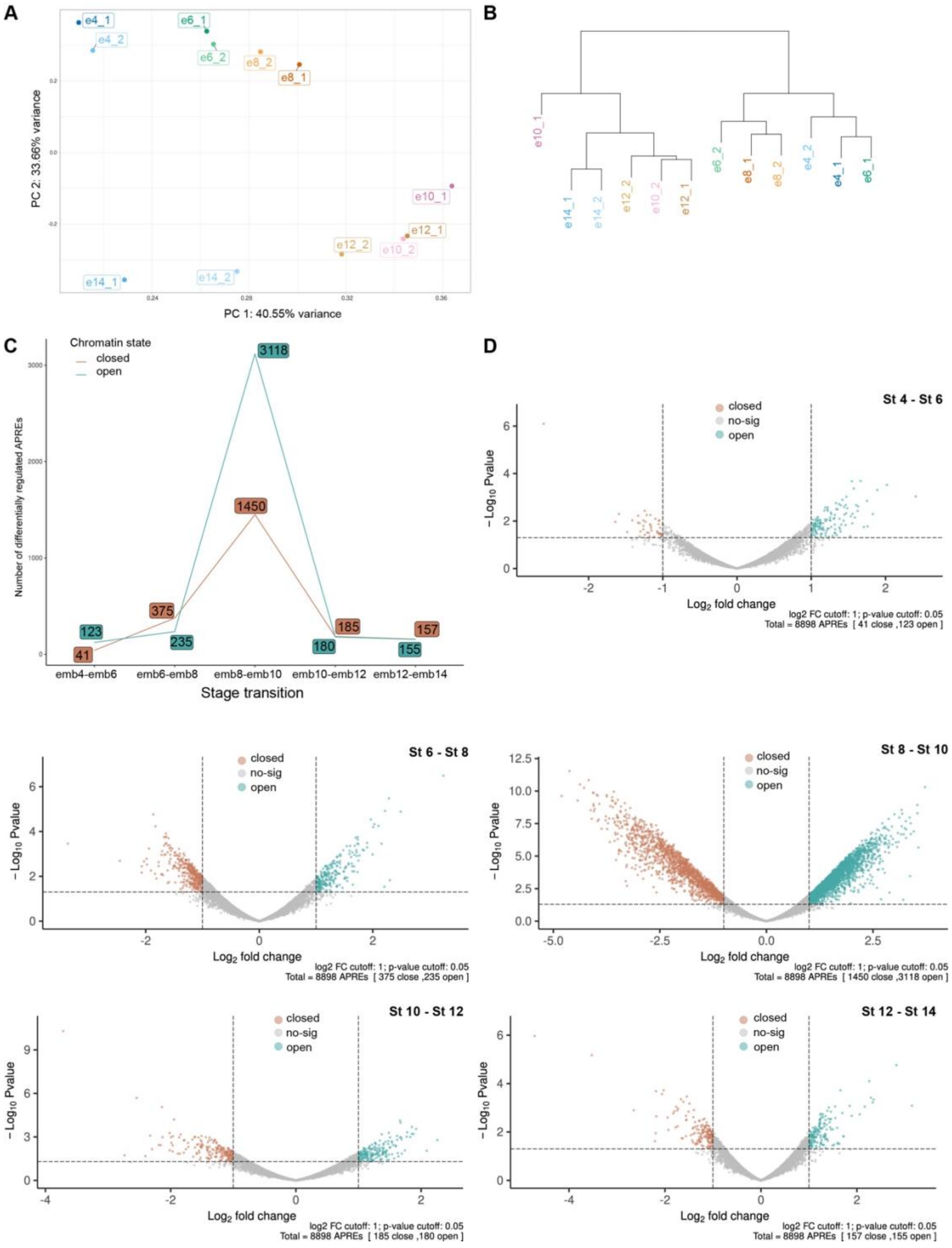

Fig. S4. PCA analysis of ATAC libraries and differential APRE accessibility. (A) PCA showing the distribution of samples across the first two principal components. **(B)** Hierarchical clustering of each sample. **(C)** Number of differentially accessible chromatin regions between the different stage transitions. **(D)** Volcano plot of all differentially accessible regions for each stage transition.

Development • Supplementary information

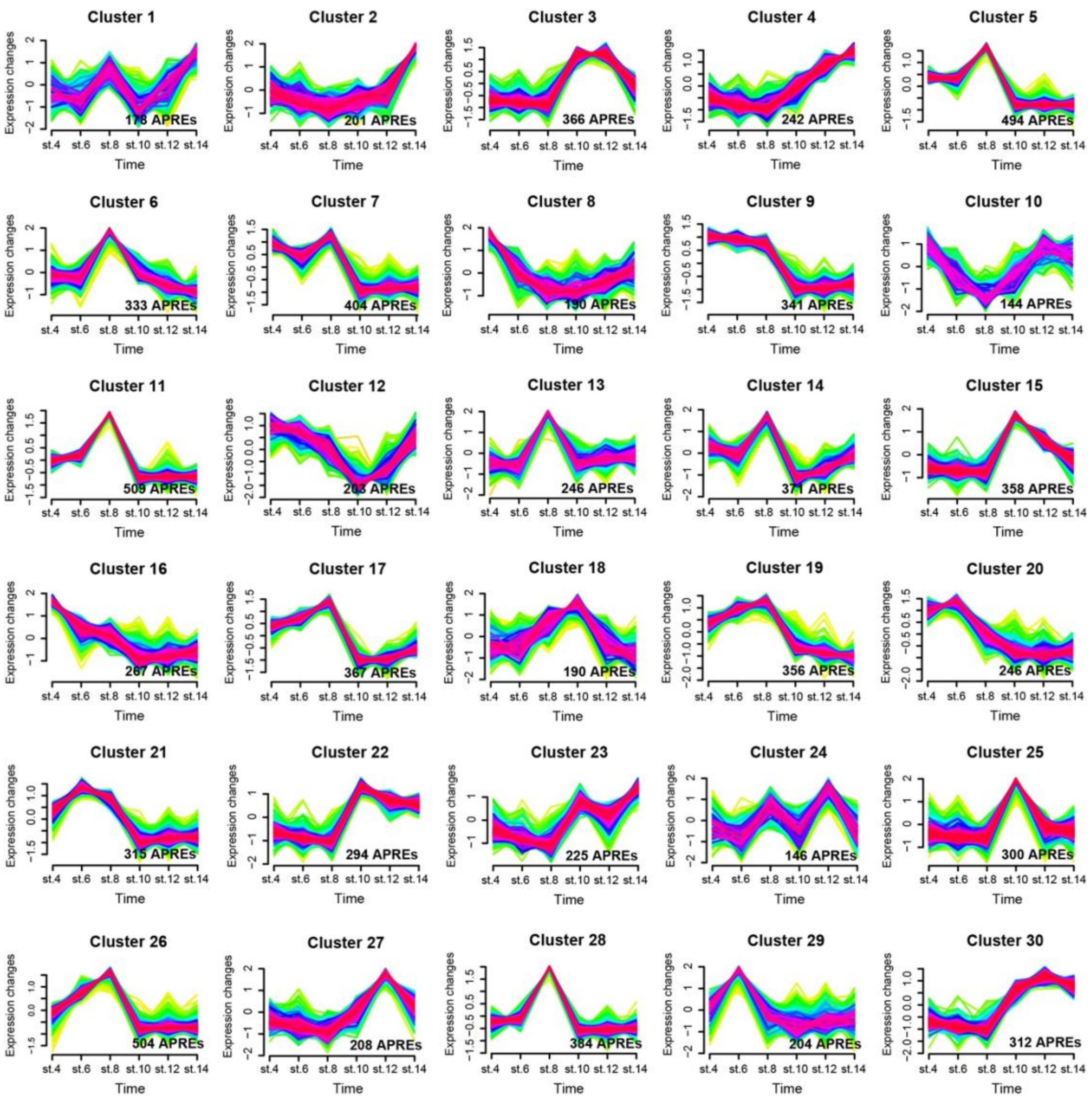

Fig. S5. Mfuzz clustering. Patterns of chromatin accessibility across different developmental stages.

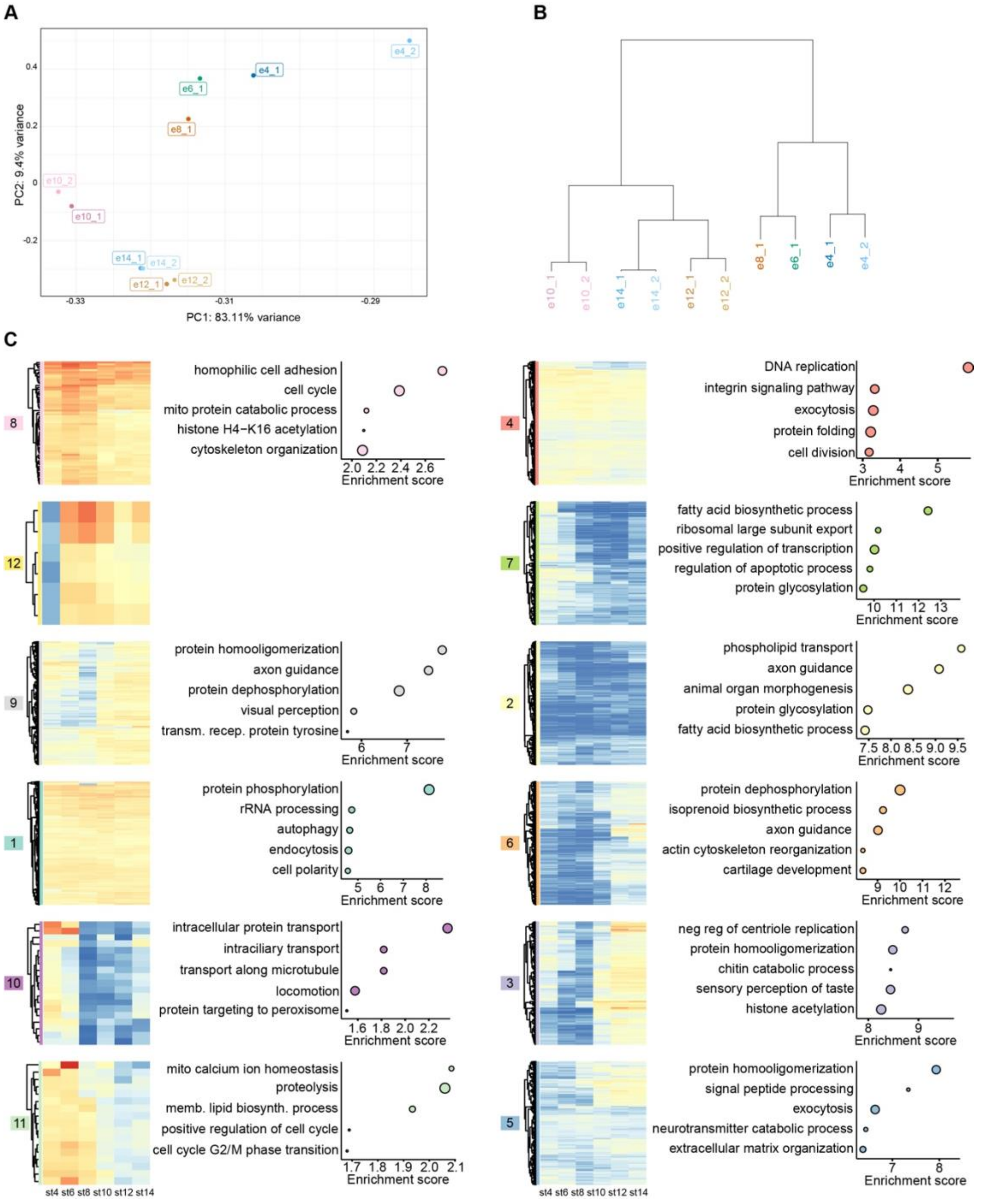

Fig. S6. PCA analysis of RNA-seq Libraries. (A) PCA displaying the distribution of RNA samples across the first two principal components. **(B)** Hierarchical clustering of RNA samples. **(C)** Gene clusters from Fig 3A with the associated GO terms. Note that due to the small number of genes included in cluster 12, there is no enriched GO terms.

Development • Supplementary information

Table S1. Counts to all the identified consensus APREs before and after normalization and mean aggregation per stage.

Available for download at
https://journals.biologists.com/dev/article-lookup/doi/10.1242/dev.203017#supplementary-data

Table S2. APREs associated to each gene and genomic zone.

Available for download at
https://journals.biologists.com/dev/article-lookup/doi/10.1242/dev.203017#supplementary-data

Table S3. Differential APRE activity data

Available for download at
https://journals.biologists.com/dev/article-lookup/doi/10.1242/dev.203017#supplementary-data

Table S4. Mfuzz output values.

Available for download at
https://journals.biologists.com/dev/article-lookup/doi/10.1242/dev.203017#supplementary-data

Table S5. Gene ontology enrichment for each Mfuzz cluster.

Available for download at
https://journals.biologists.com/dev/article-lookup/doi/10.1242/dev.203017#supplementary-data

Table S6. APREs in each cluster in the kmeans clustering.

Available for download at
https://journals.biologists.com/dev/article-lookup/doi/10.1242/dev.203017#supplementary-data

Table S7. Homer motive enrichment results for each kmeans cluster.

Available for download at
https://journals.biologists.com/dev/article-lookup/doi/10.1242/dev.203017#supplementary-data

Table S8. RNA counts before and after normalization.

Available for download at
https://journals.biologists.com/dev/article-lookup/doi/10.1242/dev.203017#supplementary-data

Table S9. Gene ontology enrichment for each gene cluster.

Available for download at
https://journals.biologists.com/dev/article-lookup/doi/10.1242/dev.203017#supplementary-data

Table S10. Homer motive enrichment results for each WGCNA module.

Available for download at
https://journals.biologists.com/dev/article-lookup/doi/10.1242/dev.203017#supplementary-data

Table S11. List of primers used for library preparation.

Available for download at
https://journals.biologists.com/dev/article-lookup/doi/10.1242/dev.203017#supplementary-data
