# Supplementary material for: Gene regulatory dynamics during the development of a paleopteran insect, the mayfly Cloeon dipterum
Source: Development. 2024 Oct 10;151(20):dev203017. doi: 10.1242/dev.203017 (PMC11491810; doi:10.1242/dev.203017)
Supplement: Supplementary file 1 [file develop-151-203017_review_history.pdf]

Gene regulatory dynamics during the development of a paleopteran insect, the mayfly *Cloeon dipterum*

Joan Pallarès-Albanell, Laia Ortega-Flores, Tòt Senar-Serra, Antoni Ruiz, Josep F. Abril, Maria Rossello and Isabel Almudi
DOI: 10.1242/dev.203017

Editor: Cassandra Extavour

Review timeline
Original submission: 1 May 2024
Editorial decision: 8 July 2024
First revision received: 24 July 2024
Accepted: 13 September 2024

Original submission

First decision letter

MS ID#: DEVELOP/2024/203017

MS TITLE: Gene regulatory dynamics during the development of a paleopteran insect, the mayfly *Cloeon dipterum*

AUTHORS: Joan Pallares-Albanell, Laia Ortega-Flores, Tot Senar-Serra, Antoni Ruiz, Josep F. Abril, Maria Rossello, and Isabel Almudi

I have now received all the referees reports on the above manuscript, and have reached a decision. The referees' comments are appended below, or you can access them online: please go to BenchPress and click on the 'Manuscripts with Decisions' queue in the Author Area.

The overall evaluation is positive and we would like to publish a revised manuscript in Development, provided that the referees' comments can be satisfactorily addressed. Please attend to all of the reviewers' comments in your revised manuscript and detail them in your point-by-point response. If you do not agree with any of their criticisms or suggestions explain clearly why this is so. If it would be helpful, you are welcome to contact us to discuss your revision in greater detail. Please send us a point-by-point response indicating your plans for addressing the referees' comments, and we will look over this and provide further guidance.

Reviewer 1

Advance summary and potential significance to field

The manuscript "Gene regulatory dynamics during the development of a paleopteran insect, the mayfly Cloeon dipterum" by Pallarès-Albanell, Ortega-Flores, and co-authors is a valuable resource for the developmental biology community interested in the evolution of animal morphology through the study of non-traditional model systems. As one of the most biodiverse groups, identifying the molecular and developmental mechanisms underpinning the diversification of insects is a central question in animal evolution. Currently, most resources, particularly those allowing the interrogation of how genomic regulation influences the deployment of developmental programmes during insect embryogenesis concentrate on a handful of systems, notably Drosophila melanogaster.

© 2024. Published by The Company of Biologists under the terms of the Creative Commons Attribution License (https://creativecommons.org/licenses/by/4.0/).

Therefore, incorporating other species, especially those occupying key phylogenetic positions, is essential. The Almudi lab has established and pioneered a new research system, the mayfly Cleon dipterum. In this manuscript, they generated ATAC-seq data for six developmental time points, which, combined with paired RNA-seq, set the ground for identifying the genes and regulatory interactions controlling the development of this insect. In a comparative framework, this will ultimately unravel the changes and novel mechanisms that promoted the fascinating diversification of insects. Openly available to the entire community through a genome browser, I foresee this will be a widely used resource by the evo-devo and developmental community.

Comments for the author

I do not have major concerns. The dataset is of good quality, and the analyses are done to a high standard. There are, however, some aspects that might improve the manuscript:

- Although commonly used, the clade Hemimetabola is paraphyletic; thus, I discourage the authors from using it (e.g. Introduction [line 91, paragraph of line 94] and Figure 1A). The system's strength is that, as members of the sister clade to Neoptera, mayflies are the key outgroup to infer what happened in the last common ancestor to the largest insect clade.

- Line 94: typo: Both holometabolous and hemimetabolous insects.

- Lines 245-252 are difficult to follow. Subdividing Fig 3A into two panels (upper with the stage clustering and lower with the gene clustering) might help. Also are these clusters obtained via k-means clustering? Explaining more about how these clusters were obtained would aid the reader. Do these follow similar dynamics to those of the dynamics APREs?

- At stage 6, the RNA-seq track shows very little expression for elav, yet there is a clear signal in the brain. A heatmap, which reflects more subtle quantitative changes in gene expression, could improve the match of HCR and RNA-seq data.

- The legends in Fig 2C and 3A need a title (what are the units?). Some text in the figures (both main and supplementary) is too small and difficult to read.

Chema Martin

Reviewer 2

Advance summary and potential significance to field

The paper presents the first genome-wide ATAC-seq analysis for a hemimetabolous insect. For their work the authors selected six embryonic stages of the mayfly Cloeon dipterum. They mapped the ATAC-seq peaks to the genome and correlated them with RNA-seq data. Different from Drosophila and more similar to Amphioxus and vertebrates the largest number of ATAC-seq peaks maps to regions distal from the transcription start site. Various clustering methods were used to analyze dynamically changing ATAC-seq tracks. Stage-specific clusters were subject to GO-term analysis of nearby candidate target genes. The enrichment of TF binding motifs way analyzed in early and late clusters. Correlations were investigated to find connections between ATAC-seq tracks and gene expression dynamics derived from RNA-seq experiments including prior established tissue-specific expression modules.
This comprehensive study of chromatin accessibility in a hemimetabolous insect will be a useful resource for comparative studies in other hemimetabolous lineages. It also can be directly re-analyzed with a focus on particular signaling pathways or developmental processes. It will help in reconstructing specific gene regulatory networks. Altogether the study will be of interest to a broad group of researchers working on the evolutions of cis-regulatory control mechanisms.

Comments for the author

The paper is will written and carefully composed. The biological conclusion drawn on the basis of the divers bioinformatic analyses are convincing and informative. I see no need for revisions.

© 2024. Published by The Company of Biologists under the terms of the Creative Commons Attribution License (https://creativecommons.org/licenses/by/4.0/).

However, I am not fully able to judge the validity of the bioinformatic methods used for cluster analyses and motif searches.

First revision

Author response to reviewers' comments

Reviewers' comments:

Note that when referring to lines in the main text, we refer to the document without tracked changes: "PallaresAlbanell_R1_nontrackedchanges".

Reviewer 1 Advance Summary and Potential Significance to Field:

The manuscript "Gene regulatory dynamics during the development of a paleopteran insect, the mayfly *Cloeon dipterum*" by Pallarès-Albanell, Ortega-Flores, and co-authors is a valuable resource for the developmental biology community interested in the evolution of animal morphology through the study of non-traditional model systems. As one of the most biodiverse groups, identifying the molecular and developmental mechanisms underpinning the diversification of insects is a central question in animal evolution. Currently, most resources, particularly those allowing the interrogation of how genomic regulation influences the deployment of developmental programmes during insect embryogenesis, concentrate on a handful of systems, notably *Drosophila melanogaster*. Therefore, incorporating other species, especially those occupying key phylogenetic positions, is essential. The Almudi lab has established and pioneered a new research system, the mayfly *Cloeon dipterum*. In this manuscript, they generated ATAC- seq data for six developmental time points, which, combined with paired RNA-seq, set the ground for identifying the genes and regulatory interactions controlling the development of this insect. In a comparative framework, this will ultimately unravel the changes and novel mechanisms that promoted the fascinating diversification of insects. Openly available to the entire community through a genome browser, I foresee this will be a widely used resource by the evo-devo and developmental community.
AR: We are grateful for the Reviewer positive comments and constructive suggestions.
Reviewer 1 Comments for the Author:
I do not have major concerns. The dataset is of good quality, and the analyses are done to a high standard. There are, however, some aspects that might improve the manuscript:

- Although commonly used, the clade Hemimetabola is paraphyletic; thus, I discourage the authors from using it (e.g. Introduction [line 91, paragraph of line 94] and Figure 1A). The system's strength is that, as members of the sister clade to Neoptera, mayflies are the key outgroup to infer what happened in the last common ancestor to the largest insect clade.
We have removed or substituted the term "hemimetabola" throughout the text and referred the comparisons to Neoptera versus Paleoptera or Pterygota versus Apterygota: Line 71: "By contrast, the remaining seventeen orders of winged insects (Fig. 1A),…" Line 75: "All insect orders shared common phases during their development…"
Line 93-94: "…collection of putative promoters and enhancers along embryogenesis of this insect."
Line 268-269: "…across embryogenesis in a paleopteran lineage using ATAC-seq approaches."
We also changed the terminology in Fig 1A and the phylogenetic diagram depicts now "Holometabola", "Neoptera" and "Pterygota".

- Line 94: typo: Both holometabolous and hemimetabolous insects.
The sentence has been changed to "All insect orders…"

- Lines 245-252 are difficult to follow. Subdividing Fig 3A into two panels (upper with the stage clustering and lower with the gene clustering) might help. Also, are these clusters obtained via k-means clustering? Explaining more about how these clusters were obtained would aid the reader. Do these follow similar dynamics to those of the dynamics APREs?

© 2024. Published by The Company of Biologists under the terms of the Creative Commons Attribution License (https://creativecommons.org/licenses/by/4.0/).

We have modified the main text and explained in more detail how the clustering and heatmap were generated:
Lines 218-223: "To do this, we measured the levels of gene expression at the same developmental stages of our ATAC-seq datasets. We focused on genes associated to the 8,898 dynamic APREs and clustered them using Euclidean distances (see methods Fig. S6 and Table S8). We first observed that these genes clustered accordingly to the six developmental stages and the "early" and "late" embryonic phases that we identified in the ATAC-seq data."

In the "methods" section:
Lines 417-426:
"**RNA-seq analysis**
For this analysis, we used the RNA-seq data generated in this project for stages 8 and 12. The remaining stages were obtained from publicly available data previously generated in the lab (PRJEB35103). Raw counts were obtained using STAR as previously described. To reduce batch effects, we normalized the data using the TMM method from EdgeR (Robinson and Oshlack, 2010) and transformed the counts to logCPM.
Heatmaps were created using the pheatmap function from the pheatmap package (Pretty Heatmaps_. R package version 1.0.12). Clustering was performed using Euclidean distance (Kolde, 2019)."

In addition, we also included a new panel A' in figure 3, Figure 3A', in which we show some representative gene clusters, to help the reader visualising the Euclidean gene clustering. Moreover, we performed an extra analysis to identify GO terms enriched in these gene clusters that it is shown together with the heatmap in Figure 3A'.
Lines 228-233: "GO terms enrichment analysis of these twelve gene clusters showed that the genes primarily expressed during early embryogenesis (clusters 11 and 7) were terms related to cell cycle progression and biosynthesis (e.g., positive regulation of cell cycle). Conversely, the genes that show increased expression in late embryogenesis (clusters 9 and 3) are associated with organogenesis (e.g., axon guidance) (Fig. 3A', Fig. S6C and Table S9)."
These new results are shown also in a new panel in Suppl. Fig. 6: Suppl. Fig. 6C and new suppl. Table: Table S9.
Note that adding this table resulted in a renaming of the posterior tables that have been modified in the text: old Table S9 is now Table S10 and old Table S10 is now Table S11.

- At stage 6, the RNA-seq track shows very little expression for elav, yet there is a clear signal in the brain. A heatmap, which reflects more subtle quantitative changes in gene expression, could improve the match of HCR and RNA-seq data.
We have included now an additional panel in figure 3, Figure 3B', which aids to visualise *Elav* expression throughout the developmental stages, in particular, stage 6, as the reviewer pointed out.

- The legends in Fig 2C and 3A need a title (what are the units?). Some text in the figures (both main and supplementary) is too small and difficult to read.
We have included now the title for the legends and increased font size of main and supplementary figures to help the reader.

Chema Martin

Reviewer 2 Advance Summary and Potential Significance to Field...
The paper presents the first genome-wide ATAC-seq analysis for a hemimetabolous insect. For their work the authors selected six embryonic stages of the mayfly *Cloeon dipterum*. They mapped the ATAC-seq peaks to the genome and correlated them with RNA-seq data. Different from *Drosophila* and more similar to Amphioxus and vertebrates the largest number of ATAC-seq peaks maps to regions distal from the transcription start site. Various clustering methods were used to analyze dynamically changing ATAC-seq tracks. Stage-specific clusters were subject to GO-term analysis of nearby candidate target genes. The enrichment of TF binding motifs were analyzed in early and late clusters. Correlations were investigated to find connections between ATAC-seq tracks and gene expression dynamics derived from RNA-seq experiments including prior established tissue-specific expression modules. This comprehensive study of chromatin accessibility in a hemimetabolous insect will be a useful resource for comparative studies in other

© 2024. Published by The Company of Biologists under the terms of the Creative Commons Attribution License (https://creativecommons.org/licenses/by/4.0/).

hemimetabolous lineages. It also can be directly re-analyzed with a focus on particular signaling pathways or developmental processes. It will help in reconstructing specific gene regulatory networks. Altogether the study will be of interest to a broad group of researchers working on the evolutions of cis-regulatory control mechanisms.

Reviewer 2 Comments for the Author
The paper is well written and carefully composed. The biological conclusion drawn on the basis of the divers bioinformatic analyses are convincing and informative. I see no need for revisions. However, I am not fully able to judge the validity of the bioinformatic methods used for cluster analyses and motif searches.
AR: We thank the Reviewer for his/her positive comments

Second decision letter

MS ID#: DEVELOP/2024/203017

MS TITLE: Gene regulatory dynamics during the development of a paleopteran insect, the mayfly *Cloeon dipterum*

AUTHORS: Joan Pallares-Albanell, Laia Ortega-Flores, Tot Senar-Serra, Antoni Ruiz, Josep F. Abril, Maria Rossello, and Isabel Almudi

I am happy to tell you that your manuscript has been accepted for publication in Development, pending our standard publication integrity checks.

Reviewer 1

Advance summary and potential significance to field

The authors adequately clarified all the raised points.

Comments for the author

Congratulations; fantastic work!
Chema Martin

Reviewer 2

Advance summary and potential significance to field

The revision addressed comprehensively all the concerns of the reviwers.

Comments for the author

The revision addressed comprehensively all the concerns of the reviwers.

© 2024. Published by The Company of Biologists under the terms of the Creative Commons Attribution License (https://creativecommons.org/licenses/by/4.0/).
